# Age-varying associations between e-cigarette use and peer use, household use, and exposure to e-cigarette commercials among alternative high school students in Southern California

**DOI:** 10.18332/tid/116412

**Published:** 2020-02-04

**Authors:** Ndifreke Etim, James Pike, Bin Xie

**Affiliations:** 1 School of Community and Global Health, Claremont Graduate University, Claremont, United States

**Keywords:** adolescents, electronic cigarette, peer influence

## Abstract

**INTRODUCTION:**

Electronic cigarettes (e-cigarettes) have rapidly become the most commonly used tobacco product among youth in the United States. Exposure to advertising, peer use, and household use, increases the risk of current e-cigarette use; however, the influence of these factors may be dynamic across adolescence. The aim of this study is to examine the age-varying associations between e-cigarette use and peer use, household use, and exposure to e-cigarette commercials among alternative high school students in Southern California.

**METHODS:**

Using data previously collected for a tobacco marketing study, we examine the age-varying associations of current e-cigarette use and three risk factors (peer use, exposure to commercials, and household use) across ages 15 to 20 years using time-varying effect modeling (TVEM). Analyses include three waves of data from alternative high school students (N=1060 students; 2036 observations).

**RESULTS:**

The probability of e-cigarette use gradually increased over the age of 15 years and then decreased by the age of 17 years for females and after the age of 18 years for males. Significant gender differences were observed between the ages of 17 and 19.5 years. Peer e-cigarette use was associated with higher odds of current e-cigarette use across all ages for females and after the age of 16 years for males. Exposure to e-cigarette commercials increased current use significantly for males between the ages of 16 and 18 years. Household use increased the odds of current use of e-cigarettes between the ages of 17 and 19 years for males and under 16.5 and over 18 years for females.

**CONCLUSIONS:**

The findings highlight the utility of TVEM in understanding the risk factors of e-cigarette use and suggest that these factors are dynamic across adolescence.

## INTRODUCTION

Electronic cigarettes (e-cigarettes) are the most common nicotine product used by adolescents and young adults in the United States^1^. In 2018, 20.8% of high school students and 4.9% of middle school students reported e-cigarette use in the last 30 days^2^. Research on the long-term health consequences of e-cigarette use is limited. However, systematic reviews have classified e-cigarettes as a hazardous product^3^. Moreover, meta-analyses suggest that youth who use e-cigarettes are more likely to use traditional cigarettes^4^, which are estimated to cause 0.48 million premature deaths and 12.7 million medical conditions each year^5^.

Alternative high school students are at a disproportionally higher risk for substance abuse than traditional high school students^6,7^. Unfortunately, this group of students is consistently excluded from important national surveys such as the Youth Risk Behavior Survey (YRBS), and have been only included in 1998, when the survey found that 64.1% of students at alternative high schools had smoked cigarettes in the last 30 days^8^. Recent studies of alternative high schools have reported prevalence of cigarette use in the last 30 days between 38.7% to 56.3%^9,10^, compared to 8.1% among traditional high school students^11^. Given that this population is at high risk for substance use, an understanding of the dynamics of social network predictors for alternative nicotine products, such as e-cigarettes, may help to inform future research and guide the development of interventions to prevent e-cigarette use among at-risk adolescents.

Prior research indicates that most smokers initiate tobacco use during adolescence, when normative and deviant behaviors are learned from elements of their social environment, including family, peers, and media^12,13^. The dynamics of smoking in social networks (i.e. closest friends, family, any household member) suggests that a social environment supportive of e-cigarette use or use of combustible tobacco is associated with greater susceptibility to and increased likelihood of using e-cigarettes or smoking^14,15^. The use of e-cigarettes by someone else in the home is associated with higher odds of current or ever use of e-cigarettes, and an increase in the susceptibility to cigarette smoking, irrespective of e-cigarette smoking status^15,16^. A growing body of evidence suggests that adolescents with family and friends who use e-cigarettes are significantly more likely to be e-cigarette users^17,18^. Studies have also established an association between exposure to e-cigarette advertising and e-cigarette initiation and use, among youth^19,20^. It has even been suggested that exposure to e-cigarettes may be more effective at attracting new users than encouraging established smokers to switch products^21^. Taken together, the supportiveness of the social environment towards e-cigarettes may be shaping lifelong behaviors among adolescents at a time when they are particularly susceptible to outside influences.

To better understand these effects, it is critical to examine whether the power of these influences changes as one ages. Previous studies on tobacco use have shown variations in these predictors across different developmental stages^18,21^. Villanti et al.^22^ found that the effect of peer smoking decreases from early adolescence to middle adolescence, while the effect of family smoking is static across each developmental stage. These researchers also found that exposure to tobacco-related media is associated with increased smoking in both early and middle adolescence. Liao et al.^18^ found that the effect of peer cigarette use was higher during junior high school than senior high school. The objective of the current study was to expand upon prior research by examining the age-varying associations between e-cigarette use and peer use, household use, and exposure to e-cigarette commercials among alternative high school students in Southern California, between the ages of 15 and 20 years.

## METHODS

### Sampling

Participants were recruited from alternative high schools that had at least 100 students and were within 100 miles of the program offices in Claremont, California. Data provided by the California Department of Education were used to identify 183 alternative high schools that met these criteria. Following approval by the Institutional Review Board, administrators at the 183 schools were contacted in a randomly selected order. The first 29 schools that agreed to participate were enrolled in the study on a first-come, first-served basis. Between 14 October 2014 and 18 May 2015, interest forms were distributed to 6870 students from the 29 schools. A total of 2726 students returned a completed form. Program staff contacted interested students and their parents or guardians. Each participant provided written consent. Parental consent was obtained for individuals under the age of 18 years. After acquiring consent, staff members arranged a date and time for each student to complete a web-based 90-minute survey.

A total of 1060 students took part in the initial assessment, which was a 15.4% response rate (1060/6870). Each student received a $45 gift card to compensate them for their time. At the 1-year follow-up assessment, 87.1% of the original cohort (923/1060) completed a web-based (892) or phone (31) survey. Each student that completed a survey received a $50 gift card. The 137 students who did not complete a follow-up assessment had failed to respond to repeated contact attempts (n=128), withdrew from the study (n=8), or were incarcerated (n=1).

At the 2-year follow-up, 81.0% of the initial cohort (859/1060) completed a survey. A total of 832 students completed a web-based survey, while 27 students completed a computer-assisted telephone interview. Each participant that completed an assessment received a $100 gift card. The 201 participants that did not complete an assessment had failed to respond to repeated contact attempts (n=187), withdrew from the study (n=9), had died (n=3), were incarcerated (n=1) or were deployed overseas after enrolling in the military (n=1). During the 2-year follow-up assessment, an effort was made to contact each of the 128 students who had failed to respond to repeated contact attempts during the 1-year follow-up assessment. None of the students returned messages delivered by email, text, phone or through social media.

Data were collected on the variables of focus in the present investigation as well as a number of additional variables beyond the scope of this study. These variables have been described in other publications^23,24^. The present focus on age-varying associations does not overlap with other work on these data.

Demographics such as sex, ethnicity and birthdate were assessed for each student. Birthdate was used to calculate the age of the student at the time of assessment, with a mean age of approximately 17.46 (SD=0.88) years at baseline. Sex was recorded according to self-report, with 50.3% reporting being male and 49.7% female. Ethnicity was recorded according to self-report, with 75.2% of the baseline sample reporting being Hispanic and 24.8% not Hispanic.

### Measures

#### E-cigarette use in the past 30 days

To quantify recent e-cigarette use, students were asked: ‘During the past 30 days, on how many days did you use electronic cigarettes, vaporizers, or vape pens?’. Response options included: 0, 1–2, 3–5, 6–9, 10–19, 20–29, or 30 days. Recent use was coded as ‘0’ if no use was reported in the past 30 days, and ‘1’ if use was reported.

#### Exposure to e-cigarette commercials

Two initial questions asked participants if they had ever seen an e-cigarette commercial on television or the internet. Frequency of exposure to e-cigarette commercials in the last 6 months was assessed by asking participants: ‘About how often did you see an electronic cigarette commercial in the last six months?’. Response options included: never, less than once a month, once a month, 2–3 times a month, once a week, 2–6 times a week, or every day.

#### Household use of e-cigarettes

A single item was used to assess the household use of e-cigarettes. Students were asked: ‘Does anyone who lives with you now use electronic cigarettes, vaporizers, or vape pens?’. Participants could respond with a yes or no.

#### Peer use of e-cigarettes

Information on peer use of e-cigarettes was obtained by asking participants: ‘How many of your four closest friends use electronic cigarettes, vaporizers, or vape pens?’. Response options included: none, one, two, three, four, or not sure. Participants who replied ‘not sure’ were recoded as ‘missing’.

### Analysis

For the current analysis, data from the three waves were combined for all participants. Only participants aged 15–20 years were included in this analysis due to low frequencies below 15 or over 20 years of age. This resulted in an analytic sample size of 2034 observations. Time-varying effect modeling (TVEM) uses all available data and accounts for within-subject correlation while addressing challenges related to unequal observations such as an unbalanced number of assessments and missing data^25^. For this analysis, observations from each participant were missing if the participants were below 15 or over 20 years of age, if the participants had dropped out of the study, or if measures of interest were not provided. Chi-squared tests or Fisher’s exact test was used to examine differences in participants with missing data due to dropping out or planned absence due to age. Males were more likely to have dropped out of the study compared to females (p=0.005). There was no difference in baseline e-cigarette use (p=0.660), exposure to e-cigarette commercials (p=0.766), peer e-cigarette use (p=0.283) and household e-cigarette use (p=0.731) between both groups.

The TVEM SAS Macro with p-spline smoothing was used in SAS v9.4 to fit a logistic age-varying effect model^26,27^. TVEM was used to estimate the prevalence rate of e-cigarette use as a function of age, and the age-varying association between e-cigarette use and each of the following variables: household use of e-cigarettes, peer use of e-cigarettes, and exposure to e-cigarette commercials. The model assumed that this change occurred smoothly over time, but did not make any parametric assumptions (e.g. linear or quadratic) about the change in effects^27-29^. Within each assessment year, all variables in the model were measured at the same time point; hence, cross-sectional relationships were estimated by the model.

First, an intercept-only model was used to estimate the rate of e-cigarette use as a function of age. Second, a model including sex as a covariate was used to examine subgroup differences in the age-varying prevalence of e-cigarette use. Third, TVEM models were run for the age-varying associations between time-varying predictors and outcomes overall. Finally, since sex was significantly related to the age-varying rate of e-cigarette use (p<0.05), TVEM models were estimated separately for males and females. All three predictors were included together in the model, so that the other two served as time-varying covariates. Ethnicity (Hispanic vs not Hispanic) was included as a time-invariant covariate in all models.

## RESULTS

**Figure 1:**
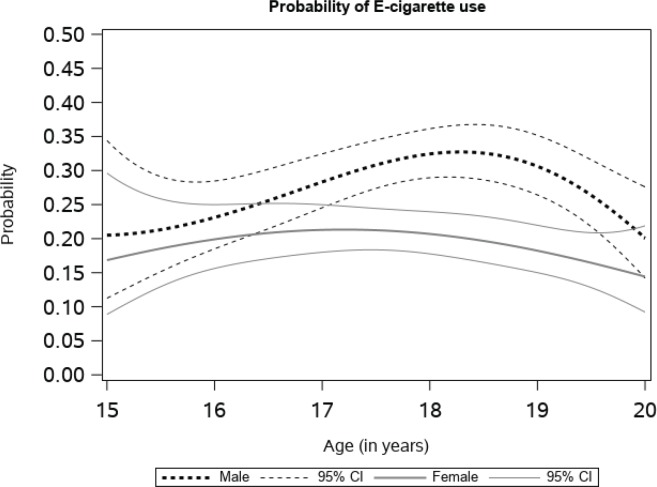
Probability of e-cigarette use by gender

Figure 1 shows the probability of e-cigarette use as a function of developmental age by sex. This curve shows the age-varying intercept transformed from the logit to the probability scale, reflecting the overall rate of e-cigarette use as a continuous function of age from 15 to 20 years. The probability of use for females was 0.17 (95% CI: 0.09–0.30) at age 15 years, rising slowly to a high of 0.21 (95% CI: 0.18–0.25) before gradually declining to 0.14 (95% CI: 0.09–0.22) at age 20 years. For males, the probability of e-cigarette use at age 15 years was 0.21 (95% CI: 0.11–0.34), followed by a gradual rise to a high of 0.33 (95% CI: 0.29–0.37) at about age 18 years and then decreasing to 0.20 (95% CI: 0.14–0.28) at age 20 years. The probabilities of e-cigarette use for males and females were significantly different after age 17 to 19.5 years, indicated by the non-overlapping confidence intervals.

### Peer use of e-cigarettes

**Figure 2:**
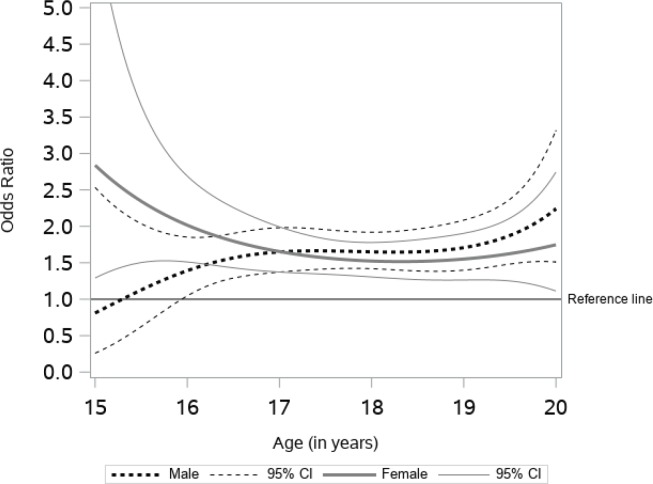
Association of peer e-cigarette use and current e-cigarette use

Figure 2 presents the age-varying association between peer e-cigarette use and current e-cigarette use. As associations are presented as odds ratios, confidence intervals not containing 1 (reference line included in plot) indicate a statistically significant association. For male students, the association was not statistically significant before age 16 years, while the association was statistically significant and stronger for females before age 16 years. At age 16 years, increasing the number of friends that use e-cigarettes by one is associated with higher odds of e-cigarette use of 1.39 (95% CI: 1.05–1.85) for males and 2.01 (95% CI: 1.51–2.68) for females. Between the ages of 16 and 20 years, this association was stable and statistically significant for both males and females, showing a similar pattern for both sexes. At age 20 years, the odds of e-cigarette use for each friend that uses e-cigarettes was 2.24 (95% CI: 1.51–3.32) for males and 1.74 (95% CI: 1.11–2.74) for females.

### Exposure to e-cigarette commercials

**Figure 3:**
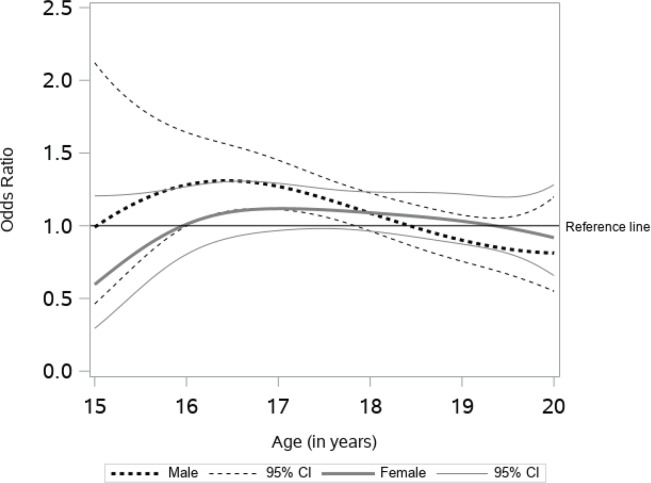
Association between e-cigarette commercials and current e-cigarette use

Figure 3 shows the age-varying association between exposure to e-cigarette commercials and current e-cigarette use. The age-varying effect model revealed that there was no statistically significant association between exposure and the current use of e-cigarettes among female participants of all ages. However, for male participants, the relationship is statistically significant between the age of 16 and 18 years. At 17 years, the odds of e-cigarette use for every unit increase in the frequency of exposure to e-cigarette commercials was 1.27 (95% CI: 1.11–1.27). This suggests a positive association between the frequency of exposure to e-cigarette commercials and e-cigarette use for males between the ages of 16 and 18 years.

### Household e-cigarette use

**Figure 4:**
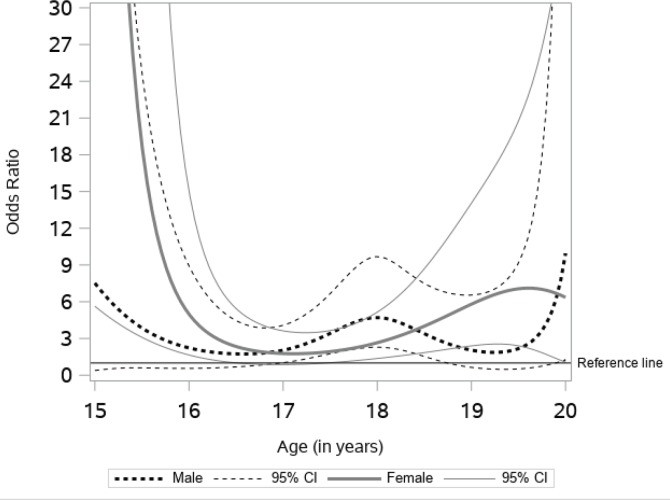
Association between household e-cigarette use and current use

Figure 4 presents the age-varying association between household e-cigarette use and current e-cigarette use. For males, the odds ratio of e-cigarette use decreases from age 15 years, reaches a low point at age 17 years, gradually increases to its peak at age 18 years, and then is followed by another wave of similar up and down fluctuation with the bottom of the curve at age 19 years. The statistical significance is sustained only during the segment of the curve between ages 17 to 19 years. The odds ratio of e-cigarette use was 2.07 (95% CI: 1.04–4.13) at age 17 years and 4.70 (95% CI: 2.28–9.57) at age 18 years. The association was statistically significant for females up to 16.5 years and after 18 years. Female students in these age groups that reported living with an e-cigarette user were more likely to currently use e-cigarettes compared to those that did not live with an e-cigarette user, with odds ratio of 4.21 (95% CI: 1.45–12.72) at age 16 years and 2.72 (95% CI: 1.39–5.30) at age 18 years.

## DISCUSSION

This study contributes to an understanding of the time-varying effects of several e-cigarette risk factors among alternative high school students. Our data suggest that the effect of such risk factors may be dynamic, not static, across adolescence to early adulthood in this sample. We found similar patterns of e-cigarette use among males and females until the age of 17 years, while after that age males had significantly higher rates of e-cigarette use. For both males and females, having friends that used e-cigarettes was associated with higher odds of current e-cigarette use. While the association remained stable for males and females between ages 16 to 20 years, the strongest association for females was before age 16 years and for males at age 20 years. The age-varying association between exposure to e-cigarette commercials was significant for males only; specifically, between the ages of 16 and 18 years. The association between household use and current use of e-cigarettes was positive across age, indicating higher odds of current use. For males, the association was significant between the ages of 17 and 19 years, while for females it was significant before 16.5 years and after 18 years.

Our findings indicate that peer e-cigarette use is an important risk factor for current e-cigarette use across the studied age groups and should be used as a potential intervention tool to reduce the increasing rate of e-cigarette use among at-risk adolescents. Specifically, our results suggest that having a friend that uses e-cigarettes is associated with higher odds of e-cigarette use across all ages for females and for males over the age of 16 years. This is consistent with previous studies on e-cigarette use and tobacco smoking that show the impact of peers on adolescent use^30-32^. Interestingly, the association was strongest for females before 16 years and strongest for males at age 20 years, highlighting two potential stages of development where intervention could be targeted. In addition, the model also suggests that the influence of close friends from adolescent years may continue into adulthood. Studies of tobacco smoking have shown that two underlying processes explain the relationship—friend selection and peer influence^33^. The findings in this study lend further support to the effect of peer influence on current e-cigarette use among youths attending alternative high schools while demonstrating that this effect remains consistent throughout the adolescent years and possibly into adulthood.

Our analysis of e-cigarette commercials suggests that the frequency of exposure to e-cigarette commercials was associated with significantly higher odds of e-cigarette use for males only and specifically between the ages of 16 and 18 years. Previous studies have documented a positive association between exposure to commercials and advertisement of e-cigarettes and the likelihood of using e-cigarettes^20,22,34,35^. However, none of these studies examined the age at which the relationship may be significant, nor did they examine the associations by sex. Adolescents are frequent users of the internet and are highly receptive to advertising. Therefore, the high amount of youth-appealing content in e-cigarette commercials^36,37^, along with frequent exposure to e-cigarette commercials, may increase adolescents’ susceptibility and acceptability of e-cigarettes—increasing the likelihood of e-cigarette use and eventually tobacco cigarette use^19,34^. Given the observed association between reported exposure to e-cigarette commercials and e-cigarette use from our study, future studies should investigate the underlying process or mechanism of this association. Also, future intervention studies are needed that examine if limiting exposure to e-cigarette commercials may be a useful tool in decreasing e-cigarette use in this specific population.

Contrary to our findings, previous studies observed stronger associations when examining advertising and the use of or susceptibility to the use of e-cigarettes^20,35,38^. The lower effect and lack of significance in this study may be due to the measure of e-cigarette commercial exposure used. Two initial questions asked participants if they had ever seen an e-cigarette commercial on television and the internet. Due to these questions, participants in our study may have indicated only the frequency of exposure from television and internet sources. Studies of traditional middle and high school students suggest that retail stores may be the main medium of exposure, with internet, television and print media being a significant source of exposure as well^19^. Thus, the magnitude of the association between e-cigarette advertising exposure and current e-cigarette use may be underestimated in this analysis.

Previous studies have found an association between household e-cigarette use and current use of e-cigarettes^15,16^. One study found that e-cigarette use by other household members may increase, by up to four times, the likelihood of current use of e-cigarettes^16^. Our findings support this reasoning, observing positive associations between exposure to household use of e-cigarettes and current use. Adolescents who have a family member using e-cigarettes in the home may obtain the device from the family member^32^. In addition, having a household member that uses e-cigarettes may provide easy access to e-cigarette devices, which may contribute to adolescent use.

Similar to peer use and exposure to e-cigarette commercials, the age-varying association between household use and the current use of e-cigarettes differed by males and females. While household use was associated with significantly higher odds of e-cigarette use between the ages of 17 and 19 years for males, it was associated with current use before 16.5 years and after 18 years, for females.

### Strengths and limitations

The findings of this study must be considered along with its limitations. First, as previously stated, because of the initial questions asking about exposure to e-cigarette commercials on television or the internet, participants may have left out frequency of exposure from retail stores, print media, billboards and other important sources besides television and the internet. This may have resulted in under-reporting of exposure to e-cigarette commercials in this sample. Second, due to the cross-sectional nature of the data, causal inferences cannot be made with confidence from the observed associations. On the association between peer use and current use, this study is unable to determine the direction of the association. It is likely that e-cigarette users were more likely to select friends who also used e-cigarettes. Similarly, e-cigarette users may have a more heightened sensitivity to e-cigarette advertising, making them more likely to recall seeing a commercial. Third, this study relies on self-reported information by adolescents, which may be subject to biases such as social desirability and recall bias. Fourth, the generalizability of the findings in this study is limited by the low response rate, only 15.4% of the alternative high school students that were contacted completed the survey. Finally, because the data from the first wave were collected in 2015, e-cigarette products, advertising and social norms regarding e-cigarettes are likely to have changed for adolescents in the intervening period. Future studies may examine if the findings in this study have changed as well.

Despite these limitations, the study provides new insight into the relationship between current e-cigarette use and risk factors across age among alternative high school students. Further, this study identifies potentially vulnerable developmental stages for male and female adolescents that may be targetable for future interventions. Future research could follow in this direction to further examine the effect of specific influences such as separating TV and internet sources of commercials, breaking down internet sources into types of social media and other online sources, and differentiating influences from different household members.

## CONCLUSIONS

E-cigarettes are now the most commonly used tobacco product among adolescents and young adults in the US^1,11^. Our findings further support the current evidence that environmental factors, such as advertising and both social and household environment, are important predictors of current e-cigarette use. Specifically, exposure to e-cigarette advertising, use by peers and use by household members were positively associated with current use among adolescents. The association was age-varying and differed for males and females. As the rate of e-cigarette use among adolescents and young adults in the US increases, peer influence may contribute to an exponential increase in e-cigarette use. Additionally, more research focusing on the social dynamics of e-cigarette use is needed to curb the growing trend.
